# Supplementary material for: The Clinicopathological Distinction between Immune-Mediated Necrotizing Myopathy and Limb–Girdle Muscular Dystrophy R2: Key Points to Prevent Misdiagnosis
Source: J Clin Med. 2022 Nov 5;11(21):6566. doi: 10.3390/jcm11216566 (PMC9655252; doi:10.3390/jcm11216566)
Supplement: Supplementary file 1 [file jcm-11-06566-s001.zip › jcm-1964334-SI/Supplementary Figures.pdf]

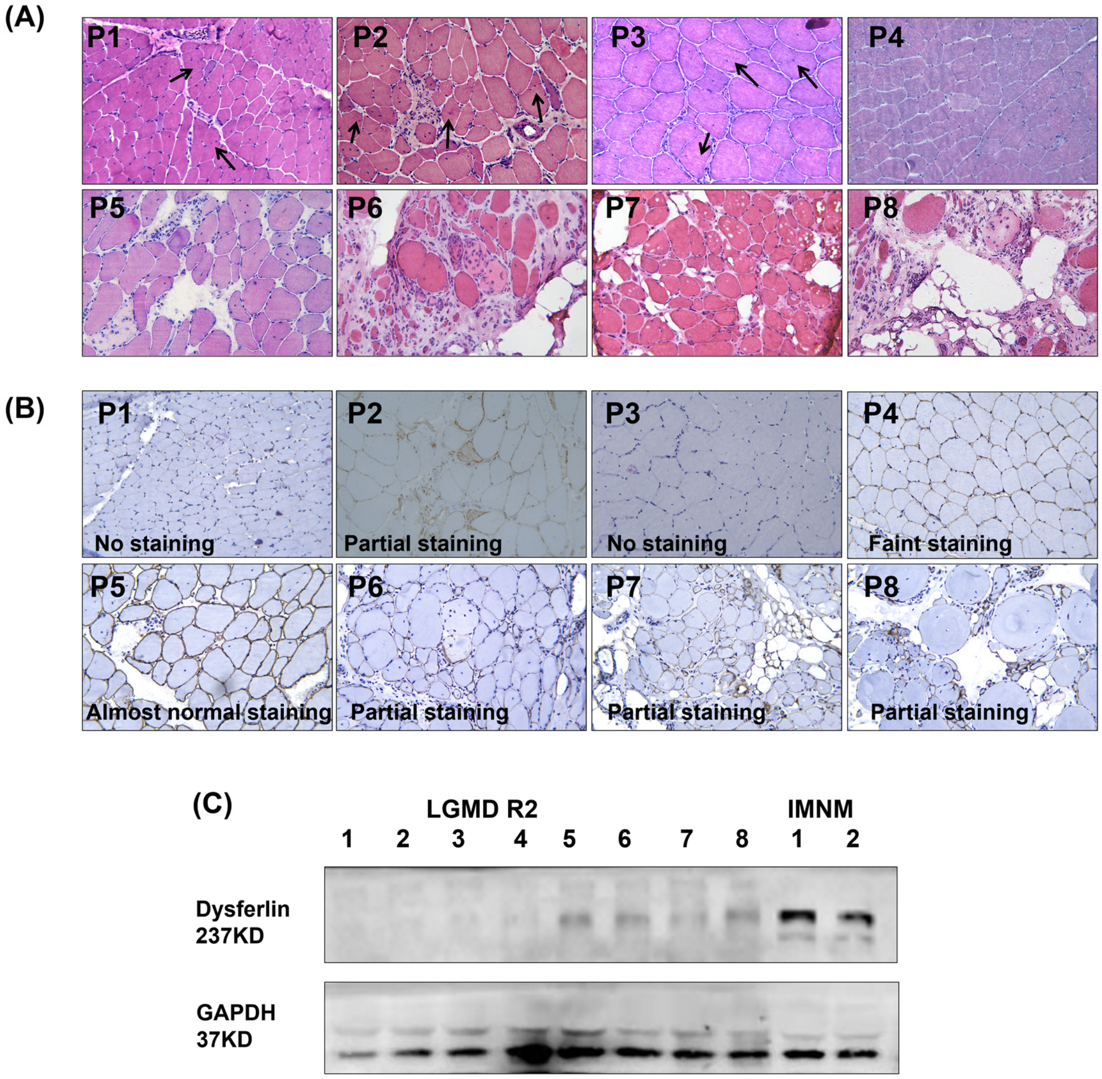

Supplementary Figure S1: Hematoxilin-eosin (H&E) staining and immunological detection of dysferlin in muscle biopsies from LGMD R2. (A) H&E staining showing scattered splitting myofibers (black arrows) in biopsies from LGMD R2 patients initially considered as IMNM (P1-P3, except P4). Prominent fat replacement or fiber size variability existed in biopsies from patients timely diagnosed as LGMD R2 (P5-P8). (B) Immunohistochemical staining for dysferlin. Images: original magnification 200 ×. (C) Western blot analysis of dysferlin protein.

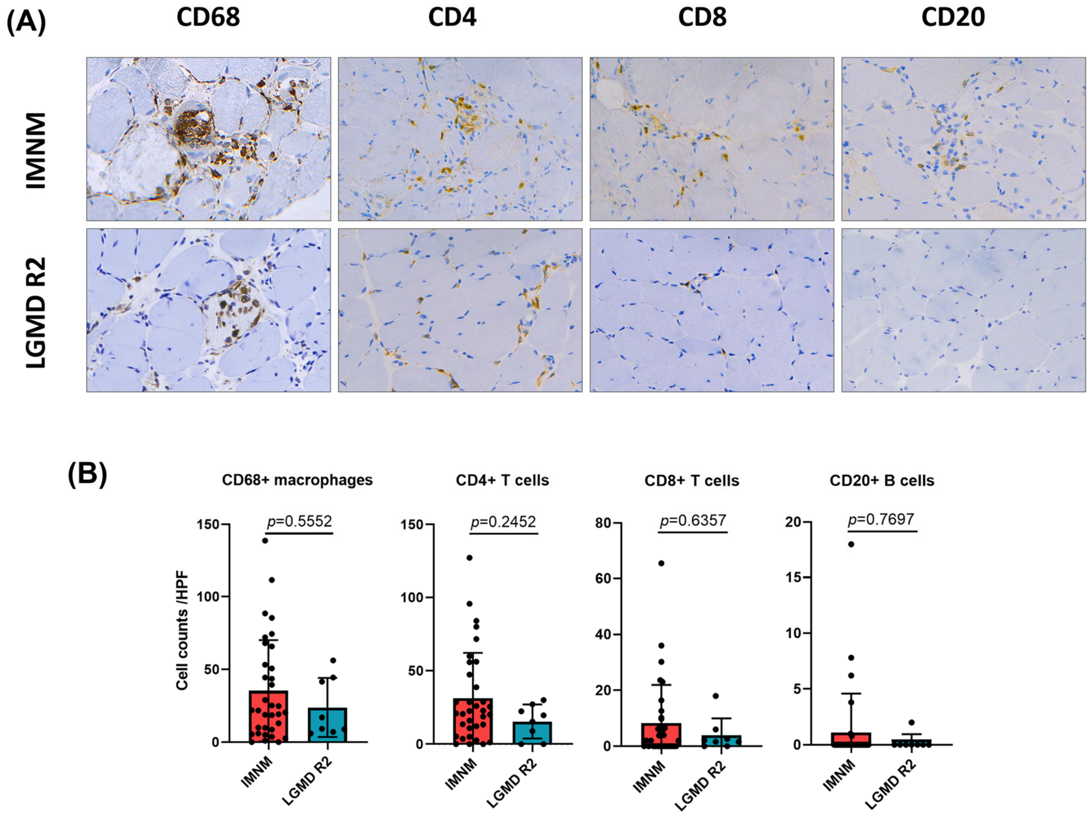

Supplementary Figure S2. Characterization of inflammatory infiltrates in IMNM and LGMD R2 biopsies. (A) Representative immunohistochemical images of D68+ macrophages, $CD4^+$ T lymphocytes, $CD8^+$ T lymphocytes and $CD20^+$ B cells. Images: original magnification 400 ×. (B) Semi-quantitative analysis of inflammatory cells.

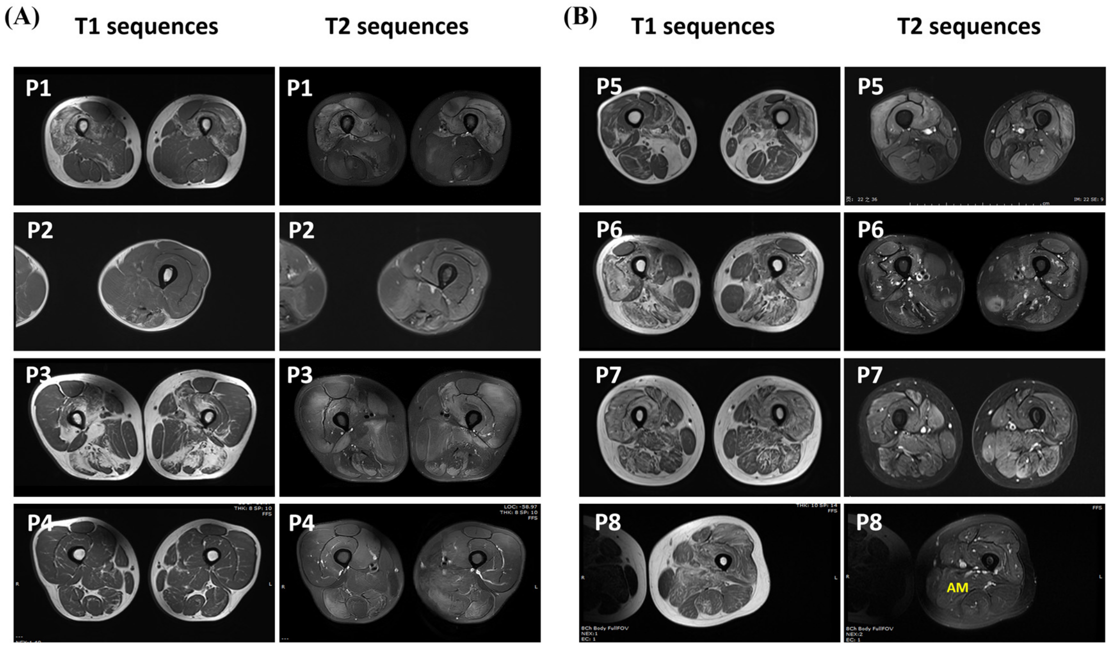

Supplementary Figure S3. Thigh muscle MRI of patients with LGMD R2. (A) Slight or no fat replacement observed on T1 images of LGMD R2 patients initially misdiagnosed as IMNM (except P3). (B) Extensive and severe fat replacement observed on T1 images of patients timely diagnosed as LGMD R2. Muscle MRI of all patients (P1-P8) showing light or no T2 hyperintensity, and the adductor magnus (AM) muscle rarely affected.
