# Supplementary material for: The Clinicopathological Distinction between Immune-Mediated Necrotizing Myopathy and Limb–Girdle Muscular Dystrophy R2: Key Points to Prevent Misdiagnosis
Source: J Clin Med. 2022 Nov 5;11(21):6566. doi: 10.3390/jcm11216566 (PMC9655252; doi:10.3390/jcm11216566)
Supplement: Supplementary file 1 [file jcm-11-06566-s001.zip › jcm-1964334-SI/Supplementary Table S1.pdf]

Supplementary Table S1. Demographic features and pathogenic mutations in patients with LGMD R2.

**Patients**	**1**	**2**	**3**	**4**	**5**	**6**	**7**	**8**
Age of onset (years)	32	30	31	26	57	32	26	24
Gender	Female	Male	Male	Male	Male	Male	Female	Female
Family history	No	No	No	No	Yes	No	No	No
MSAs or MAAs	（-）	Unknown	（-）	（-）	（-）	（-）	（-）	Unknown
Misdiagnosed	Yes	Yes	Yes	Yes	No	No	No	No
DYSF mutations	c.2646-1 G>A	c.2977 T>C	c.3113 G>A<br>c.4863 C>A	c.5512 G>A	c.4714 C>T,<br>c.1393 G>C	c.5626 G>A	c.2125 C>T<br>c.5626 G>A	c.1141_1142insG

Abbreviations: LGMD R2, limb-girdle muscular dystrophy R2; MSAs, myositis specific antibodies; MAAs, myositis associated antibodies; IHC, Immunohistochemistry.
